# Methodology to Produce Specimen-Specific Models of Vertebrae: Application to Different Species

**DOI:** 10.1007/s10439-017-1883-8

**Published:** 2017-07-25

**Authors:** Fernando Y. Zapata-Cornelio, Gavin A. Day, Ruth H. Coe, Sebastien N. F. Sikora, Vithanage N. Wijayathunga, Sami M. Tarsuslugil, Marlène Mengoni, Ruth K. Wilcox

**Affiliations:** School of Mechanical Engineering, Institute of Medical and Biological Engineering, University of Leeds, Leeds, LS2 9JT UK

**Keywords:** Bone elastic modulus, Image-based model, Sensitivity analysis, Finite element analysis, *In silico* models

## Abstract

Image-based continuum-level finite element models have been used for bones to evaluate fracture risk and the biomechanical effects of diseases and therapies, capturing both the geometry and tissue mechanical properties. Although models of vertebrae of various species have been developed, an inter-species comparison has not yet been investigated. The purpose of this study was to derive species-specific modelling methods and compare the accuracy of image-based finite element models of vertebrae across species. Vertebral specimens were harvested from porcine (*N* = 12), ovine (*N* = 13) and bovine (*N* = 14) spines. The specimens were experimentally loaded to failure and apparent stiffness values were derived. Image-based finite element models were generated reproducing the experimental protocol. A linear relationship between the element grayscale and elastic modulus was calibrated for each species matching* in vitro* and *in silico* stiffness values, and validated on independent sets of models. The accuracy of these relationships were compared across species. Experimental stiffness values were significantly different across species and specimen-specific models required species-specific linear relationship between image grayscale and elastic modulus. A good agreement between* in vitro* and *in silico* values was achieved for all species, reinforcing the generality of the developed methodology.

## Introduction

Over the last decade, the use of specimen- and subject- specific finite element models of spinal vertebrae has become more widespread to evaluate fracture risk^14^ and the biomechanical effects of diseases and therapies.^21^,^27^,^45^ These models are usually based on computed tomography (CT) or micro-CT data, enabling both geometrical and material property information to be derived from the images. In many cases, the elastic modulus is derived on an element-by-element basis using the image data within each element region.

For continuum-level models, where the element sizes are larger than the individual trabeculae, a number of approaches have been adopted to determine the elastic modulus and other material properties from the image data.^30^ In some cases, an average grayscale is determined from the voxels within the element volume and the elastic modulus is derived by assuming a relationship to this grayscale value (i.e. by assuming that the modulus is related to the bone density and the density related to the grayscale).^6^,^9^,^18^,^23^,^44^,^48^ In other cases, the trabecular architecture and bone volume fraction within each element volume are used to derive the elastic behaviour, which may include anisotropic effects based on the fabric tensor.^5^,^35^ Regardless of the method used, the relationships between the image information and the material parameters need to be derived. Many of these relationships originate from experimental tests on bone samples, whilst some have also been determined by reverse engineering sets of vertebral finite element models to fit corresponding sets of experimental test data.^20^ A number of different forms of equation have been proposed, but for vertebral bone under physiological loads, linear relationships have been shown to be as accurate as non-linear relationships,^48^ provided the associated constants have been optimally adjusted.

Experimentally, a wide range of animal models are used in bone research to investigate disease progression or the effect of interventions including (but not limited to) rodents (mouse and rat^3^,^38^,^42^,^46^), rabbits,^2^,^15^,^16^,^43^ dogs,^8^,^19^,^22^,^25^ pigs,^37^,^41^,^47^ sheep^12^,^26^,^33^,^49^ and goats.^7^,^24^,^51^ Peric *et al.* 36 summarised the similarity of animal models to human bone based on macro- and micro-structure, composition and remodelling, and placed the pig model as the closest animal (non-primate) model to human bone, followed by dogs and sheep. Rodents and rabbits on the other hand, scored low in similarities related to the macrostructure and composition, which is not surprising as both species lack a Haversian system, have permanently open growth plates and their size and shape differ from human bones.^36^ Similar animal models have been reported in the use of spinal research,^11^ with bovine, ovine and porcine being amongst the commonest models used.^1^

Specimen-specific finite element models have therefore been based on a number of different species, to allow direct comparison with experimental data and to assess variability or the potential for remodelling. Because of the variation in bone mineral density across species, and the differing ages of animal used, it is likely that the material properties for bone derived from CT images require calibration for each different species. Although finite element studies of different species have been developed, there has yet to be a comparison across multiple species to evaluate how these relationships differ.

The aim of this study was therefore to derive species-specific modelling methods and compare the accuracy of image-based finite element models of vertebrae across species.

Combined experimental and computational approaches previously described^44^,^45^,^48^ were used to develop specimen-specific finite element models of bovine, ovine and porcine vertebrae. The models of each species were divided into calibration and validation sets and the calibration sets used to derive a linear relationship between the element grayscale and elastic modulus. The parameter values and accuracy of the relationships were then compared across species.

## Materials and Methods

### Specimen Preparation, Imaging and Mechanical Testing

Mature ovine (3–5 years old), juvenile porcine (24–26 weeks old) and sub-adult bovine (2–2.5 years old) spines obtained from a local abattoir were used in this study. The specimens were harvested to isolate the bone from other tissues, yielding the following: thirteen ovine vertebrae coming from the cervical (*N* = 4) and thoracic regions (*N* = 9), twelve porcine vertebrae from the thoracic (*N* = 7) and lumbar (*N* = 5) regions and fourteen bovine vertebrae from the coccygeal region (*N* = 14).

The samples were mounted in PMMA cement endcaps to enable flat surfaces on which to apply load.^45^ A delrin fiducial marker was incorporated in the endcap to locate the position of the applied load. The ovine and porcine specimens were imaged using a *µ*CT scanner (*µ*CT, Scanco Medical AG, Switzerland) at an isotropic voxel size of 73.6 *µ*m, energy settings 114 *µ*A, 70 kVp and 300 ms exposure time. Bovine specimens were imaged using a HR-pQCT (XtremeCT, Scanco Medical AG, Switzerland) at an isotropic voxel size of 82 *µ*m, energy settings 900 *µ*A, 60 kVp and 300 ms exposure time. Each specimen was axially compressed in a material testing machine (Instron 3365 with a 10kN load cell, Instron, UK). The load was applied to the specimens* via* a stainless steel ball and loading plate, enabling the upper endplate to rotate. A pre-load of 50 N was initially applied, followed by cyclic loading with a maximum load of 300 N. The specimens were then loaded at rate of 1 mm/min and compressed until failure or reaching the safety limit of the load cell (9.8 kN). Load–displacement data was used to derive the maximal experimental stiffness of each specimen, calculated as the largest slope over a moving average of 0.6 mm aperture.

### Finite Element Modelling

All specimens were modelled with a specimen-specific approach. The geometry of each specimen was built from the 3D scan data. The CT images were truncated to remove any negative values, and the result was consistently normalised to a 0–255 grayscale (8-bit) using a bespoke script (MATLAB R2014b, The MathWorks, Inc., Natick, MA, US). Images obtained from the HR-pQCT were converted to *µ*CT equivalent images (see Appendix). The images were down-sampled to an isotropic 1 mm resolution, using a partial volume effect algorithm, and segmented to isolate the bone from the cement endcaps in Simpleware ScanIP v7.0 (Synopsys, Mountain View, USA). Morphological operations were used to produce continuum-level masks. The segmented images were meshed with a mix of linear tetrahedral and hexahedral elements of uniform size matching the down-sampled voxel size, yielding models with 79–515 thousands elements. The bone tissue was modelled with Hooke elasticity, using element-specific elasticity moduli ($E_{ele}$) dependent of the average grayscale value for the element ($GS_{ele}$):

1 $$E_{ele} = \alpha GS_{ele} (GPa)$$

where *α* is a conversion factor determined separately for each species.^44^,^45^,^48^ A Poisson’s ratio of 0.3 was used. The cement was modelled with Hooke elasticity with a modulus of 2.45 GPa and Poisson’s ratio of 0.3.^45^

Boundary conditions replicating the experimental tests were applied: the bottom surface of the lower endcap was clamped and a rigid plane tied to the upper surface of the upper endcap was defined to model the loading plate. A 1 mm translation in the axial direction was applied to the rigid plane centred on the location of the load application marker; translations in the other directions were restricted and rotations were kept free to replicate the experiment.

Within each species, the specimens were arbitrarily divided into two groups. The first group was used for calibration of *α* for each species (porcine and ovine *N* = 6; bovine *N* = 8 specimens), the second group for validation (porcine and bovine *N* = 6; ovine *N* = 7 specimens). A golden section search scalar optimisation process using the Brent method^4^ was used to derive the conversion factor *α* on the calibration group. The opti4Abq toolbox^28^,^29^ using the Brent method implementation in SciPy (Python Software Foundation, v2.7, www.python.org) was used in this work. The objective function was the root mean square normalised difference (RMSE) between experimental specimen stiffness and the corresponding finite element stiffness. The optimisation process was terminated when the objective function or its variation reached a threshold set at 0.1. All finite element analyses were Non-Linear quasi-static and run in parallel with Abaqus 6.14 (Simulia, Dassault Système, London, UK). Models were run on a standard desktop computer and each model solved under 5 min.

### Morphology

The degree of anisotropy (DA), trabecular orientation and bone volume fraction (BV/TV) were calculated for six specimens of each species using BoneJ 1.4.2^10^ together with Fiji/ImageJ 1.51 g.^39^ A region of interest (ROI) was selected by fitting the largest possible cylinder within the trabecular bone between the two endplates, with its axis parallel to the superior/inferior axis. Trabecular orientation was calculated as the deviation (in degrees) of the MIL fabric tensor^17^ principal eigenvector with respect to the superior/inferior axis.

### Statistical Analysis

After testing for normality and outliers with a Shapiro–Wilk test, the experimental stiffness values, degree of anisotropy, trabecular orientation and BV/TV values were compared between species using a Kruskal–Wallis test and post hoc Wilcoxon signed-rank test. The agreement between the *in silico* predicted stiffness values and the *in vitro* measured stiffness values was assessed using concordance correlation coefficients (CCC). All statistical analysis were performed using statistical software R.3.2.3 (R foundation for statistical computing, Vienna, Austria).

The data associated with this paper (*µ*CT images, mechanical testing results, model input files, and all processed outputs) are openly available from the University of Leeds Data Repository.^50^

## Results

### Experimental Stiffness

**Figure 1:**
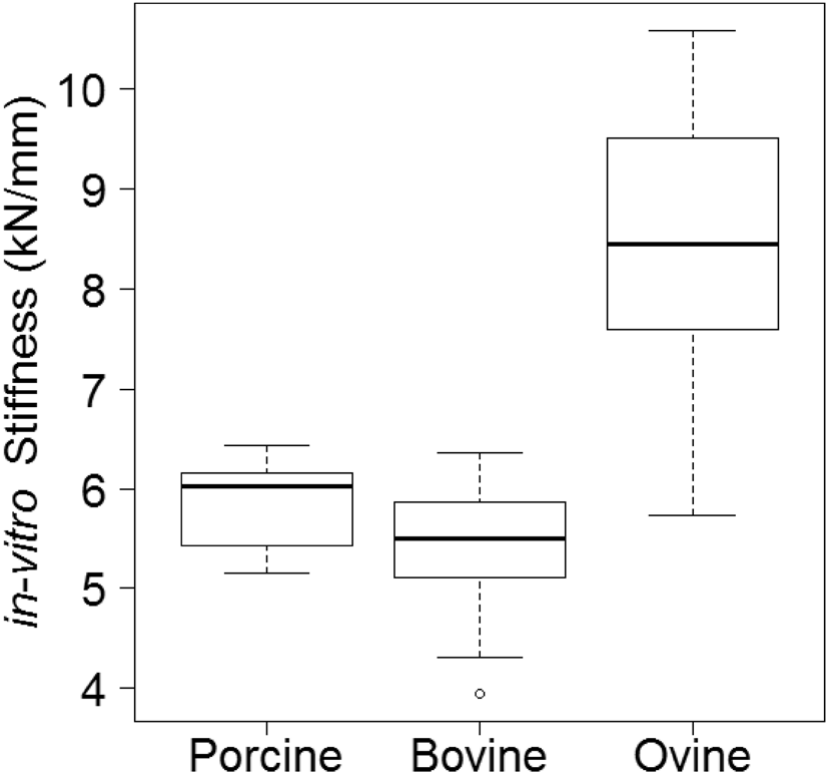
Experimental stiffness values (kN/mm) for all specimen groups.

**Figure 2:**
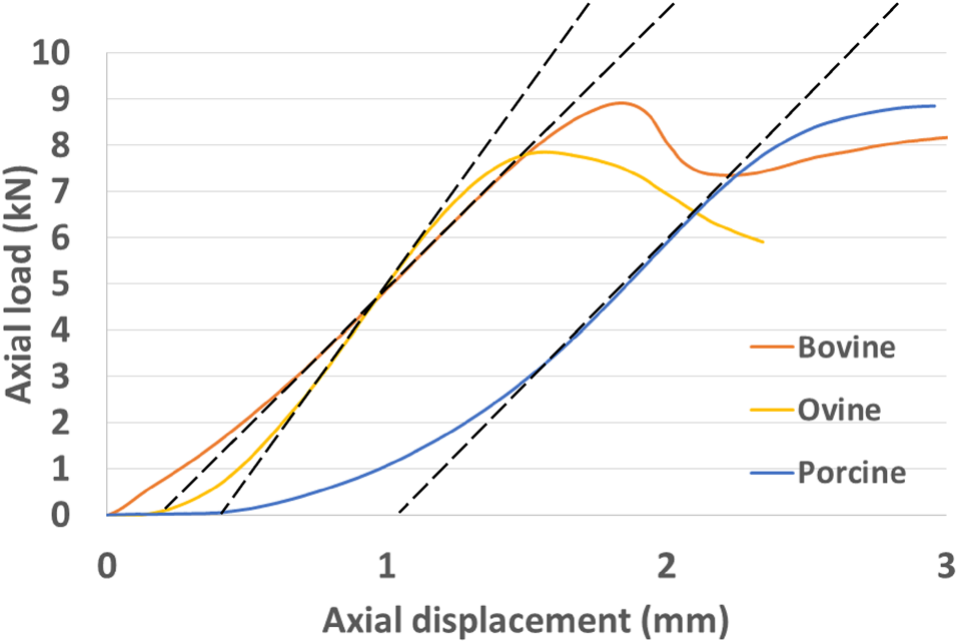
Typical examples of experimental load–displacement curves for all species showing the regions of greatest slope from which the stiffness values were calculated.

A statistically significant difference in *in vitro* stiffness values was observed between species (*p* = 6.38 × 10^−6^), for all paired test (porcine vs. bovine, *p* = 0.046; porcine vs ovine, *p* = 2.57 × 10^−5^; bovine vs ovine, *p* = 1.19 × 10^−6^). The lowest *in vitro* stiffness observed was for the bovine specimens (mean 5.3 kN/mm, st.d. 0.7 kN/mm), followed by porcine (mean 5.8 kN/mm, st.d. 0.42 kN/mm) and ovine (mean 8.4 kN/mm, st.d. 1.34 kN/mm) specimens; see Fig. 1. The load–displacement curves for typical specimens are shown in Fig. 2. Porcine specimens showed a clear non-linear behaviour with a marked toe-region while bovine specimens exhibited relatively linear behaviour before collapse.

### Computational Results

**Figure 3:**
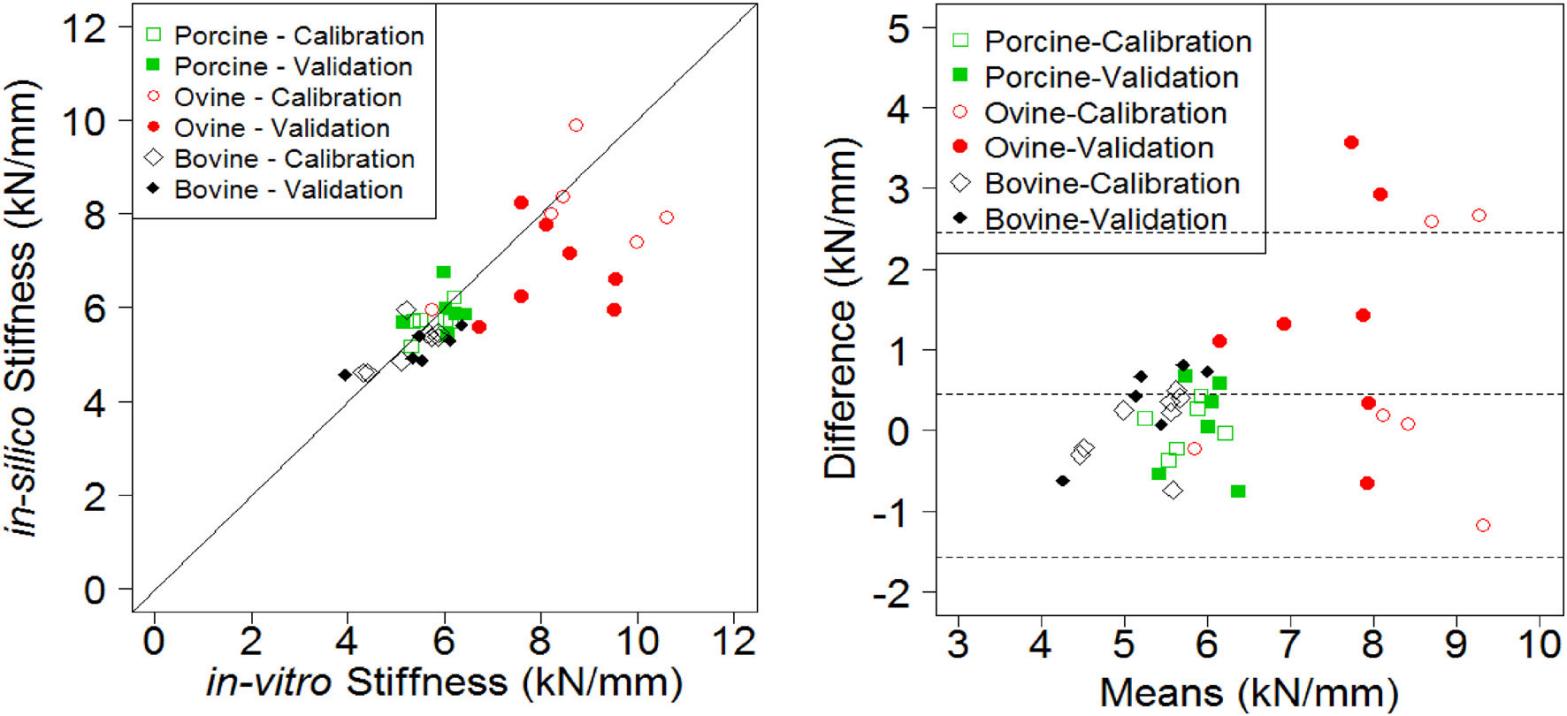
Bland-Altman plot of the *in silico* vs. *in vitro* apparent stiffness results.

For the imaging conditions and computational methodology presented, the optimisation yielded converged conversion factor *α* values of 0.00726 GPa for the bovine tissue, 0.00904 GPa for porcine and 0.00971 GPa for ovine. The stiffness RMSE was below 16% for each calibration group (Table 1), with the highest local relative error (one ovine specimen) being 25.9% (see Fig. 3). Using each of the species-specific conversion factors on the corresponding validation sets yielded a RMSE below 22% for all groups, with the highest relative error for an individual specimen (ovine) being 37.5%.

**Table 1:** RMS error yielded by the optimisation function for each species.

Specimen	Calibration (%)	Validation (%)
Porcine	4.8	9.2
Bovine	7.7	11.3
Ovine	16.0	21.7

Good agreement between *in vitro* and *in silico* stiffness values was achieved for bovine specimens (CCC = 0.6193), while it was lower for ovine (CCC = 0.2356) and porcine (CCC = 0.3902) specimens.

### Morphological Assessment

The results of the morphology analysis are presented in Table 2, with significant variation between species in the DA (Kruskal–Wallis test, *p* = 4.18 × 10^−5^; porcine vs. bovine, p = 1.55 × 10^−4^; porcine vs. ovine, *p* = 1.55 × 10^−4^; bovine vs. ovine, *p* = 3.10 × 10^−4^), and the ovine vertebrae showing the highest anisotropy. There was a significant difference between species in the BV/TV values (Kruskal–Wallis test, *p* = 0.0066). The post hoc test revealed that this difference was observed only for the porcine vs. bovine pair (Wilcoxon test, *p* = 0.0029).

**Table 2:** Summary of the morphological results; 2D and 3D view of the ROI of the analysed trabecular bone for each species.

Species	DA Mean ± st.d.	BV/TV Mean ± st.d.	Sample 2D	Sample 3D
Porcine	0.321 ± 0.067	0.457 ± 0.064		
Bovine	0.591 ± 0.074	0.345 ± 0.053		
Ovine	0.787 ± 0.03	0.402 ± 0.052		

The ROI was selected by fitting the largest possible cylinder within the trabecular bone between the two endplates

For all specimens, the trabecular orientation was mainly aligned with the superior/inferior axis, (mean deviation of 2.2°, st.d 1.1° for ovine; mean 3.6°, st.d 1.6° for bovine; mean 8.4°, st.d 10.8° for porcine). There was a significant difference between species in the trabecular orientation results (Kruskal–Wallis test, *p* = 0.01231) and the post hoc test revealed that this difference was observed only for the porcine vs. ovine pair (Wilcoxon test, *p* = 0.004662) and the ovine vs. bovine pair (Wilcoxon test, *p* = 0.04988). Table 2 shows examples of the bone plugs analysed in 2D and 3D views.

## Discussion

A previously developed methodology was used for the generation of specimen-specific finite element models of vertebrae from *µ*CT data, and for the determination of the grayscale to Young’s modulus conversion factors for each species. The models were validated against corresponding experimental compression tests for porcine, ovine and bovine tissue. Results showed significant differences between each of the tested species, in their experimental behaviour, in the assessment of their morphology, and in the conversion factor needed to produce finite element models with good agreement.

The low error on the stiffness values for the validation sets was of the same order of magnitude as for the calibration sets, it is thus fair to conclude that, for the tested conditions and *in silico* methodology, this is a valid modelling approach to represent the vertebral apparent stiffness in axial compression.

### Inter-Species Variation

All tested groups showed a good agreement between *in silico* and *in vitro* stiffness values, however a varying degree of concordance was observed. This may be a reflection of the variation and spread of the stiffness values seen within each of the different groups, where the ovine specimens have the largest range of values and the largest RMSE.

Of the many factors that could explain the difference seen between the conversion factors for different species, most can be grouped around properties either not captured in *µ*CT scans or not present within the finite element models. Of the first type, the *µ*CT does not allow for any distinction in the type of interstitial marrow or other tissues. It is likely that the preparation protocol, from the moment of slaughter in the abattoir to the testing of the specimen in the laboratory, generates different states of these tissues, with more or less clotted blood within the samples, and more or less marrow content depending on the species. Other tissues include the growth plate, a cartilaginous tissue softer than bone, of which the size depends on the maturity level of the specimens used. The interstitial tissue and growth plates, however, are averaged either in the trabecular or cortical space grayscale value and its stiffness variation is not at all accounted for in the computational models. Finally, the ovine specimens showed the largest error and also presented with the largest range of stiffness values in the experimental data. This is mainly due to the difference in size between the cervical (*N* = 4) and thoracic (*N* = 9) vertebrae, for which the cervical vertebrae exhibited a lower stiffness. It is possible that considering different conversion factors for different vertebral level to account for differences in the underlying tissue properties would improve the error.

Regarding the properties not included in the computational models, some of the details relating to the internal geometry of the vertebrae are lost when down-sampling the images. Information such as the trabecular direction, the ratio of cortical bone to trabecular bone (the cortical shell thickness), and to some extent the bone volume fraction (BV/TV) and level of mineralisation, is all merged into the grayscale of the image underlying each element. Interspecies variation in these properties may be a factor in the variation of the conversion factor across species. In particular, the degree of anisotropy (DA) varied significantly between species, with the porcine bone containing the most isotropic trabecular structure and the ovine tissue containing the most anisotropic trabeculae. This increased trabecular orientation, with more trabeculae aligned axially, gives an increased stiffness in axial compression for the ovine vertebral bodies, which also shows a higher conversion factor. However, given the lack of correlation between the DA and the conversion factor, other properties may explain the variation across species, such as BV/TV. For instance, the reduced bone volume fraction for bovine vertebrae is due to areas devoid of trabeculae within the vertebral body which possibly explains its low value for the conversion factor, despite the relative alignment of its trabecular network with the loading direction.

### Imaging Considerations

The conversion factor α calculated for each species is valid only when used with 8-bit grayscale images generated from the *µ*CT 100 and the scanning settings described in the specimen preparation section, thus making the value of α dependent on the equipment and settings used to scan the specimen. A calibration method was developed to enable the conversion of 8-bit images generated from HR-pQCT to *µ*CT equivalent images, with an acceptable degree of accuracy (see Appendix). This provides a framework to reuse our method to develop *in silico* vertebrae tests regardless of the equipment or scanner settings (i.e. resolution) when used within reasonable limits.

## Limitations and Challenges

The finite element models were built using linear material models and non-linear geometrical effects. Using such material models allows only a representation of the linear part of any load–displacement behaviour. Within human vertebrae however, the linear portion of the *in vitro* load–displacement curve corresponds to the physiologically relevant loading regime.^31^,^32^,^34^,^40^ The material models does not allow us to represent the full non-linear experimental behaviour observed for all species in this study. There is no clear indication whether the non-linearity at low displacements is mainly a material non-linearity or whether it is linked to the experimental setup and the interactions between different components.

The results of this study suggest that there is not a universal conversion factor that can be applied to derive vertebral bone elastic modulus from the image grayscale. Across species tested, the conversion factor increased with increasing vertebral stiffness, but the relationship was not linear and the number of species is not enough to draw a concrete conclusion. Higher levels of accuracy in the FE predictions could be achieved by further narrowing the specimen choice (e.g. to a particular spinal region), but would clearly then limit the application of the derived conversion factor. The values presented here are valid only for ovine tissue from mature animals, porcine tissue from juvenile animals and bovine tissues from sub-adult animals, and for vertebral bone submitted to axial compression, and imaged and meshed following a prescribed methodology. In particular, the tissue preparation will affect the results, as will the image resolution and imaging method. These will change how the bone density is represented in the images that the models are built from. Moreover, the type and size of mesh elements in the computational models will influence the results. The methodology however can be extended to other tissue preparation and model development steps with appropriate calibration procedures.

## Conclusion

This study showed that the methodology for generating specimen-specific vertebral finite element models previously developed for a given species can be translated to other species, but without a universal value of grayscale to elasticity modulus conversion. This single parameter includes a large range of properties and varies from species to species. However, when care is taken to ensure specimen preparation reproducibility, and phantoms are used to convert image data from one *µ*CT system to another, then there is no reason why the conversion factor established in a given *in vitro* and *in silico* environment should not be re-used for other tests. In particular, in the present case of vertebral bone or models that incorporate several vertebral joints can use the conversion factors derived in this work.

## Figures and Tables

**Figure 4:**
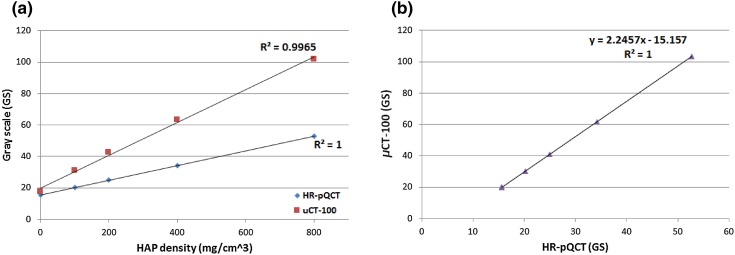
(a) Relation between average GS and known-density from phantom scanned in both CT equipment. (b) Linear relationship found between GS from HR-pQCT and *µ*CT.

## Appendix: Image Conversion: Calibration Methodology

### Introduction

It is known that the range of grayscale values of an image generated from CT equipment is machine dependent.^13^ For this study, a calibration methodology was generated for the conversion of the images generated from a HR-pQCT (XTremeCT, Scanco Medical, Switzerland) to images that could be comparable to a set of images from a *µ*CT system (microCT100, Scanco Medical, Switzerland).

### Methodology

A *µ*CT phantom with five known density zones (0–800 mg HA/cm^3^) was scanned in both machines and the images were converted to 0–255 grayscale following the methodology described in this work. Each density zone was segmented for both sets of images and an average grayscale value for each density was calculated in an image processing package (Simpleware ScanIP v7.0, Synopsys, Mountain View, USA). A linear relation between average grayscale values and the densities was derived for each imaging facility. From this, a linear relation between the original HR-pQCT image and the equivalent *µ*CT one was derived, enabling the conversion of HR-pQCT images to *µ*CT images (*µ*CT equivalent or *µ*CT-E images). Pearson’s coefficients were calculated to verify the linearity of the density/grayscale data.

In order to validate this methodology, three bovine vertebrae from the coccygeal region were scanned in both CT machines and the HR-pQCT images were converted to *µ*CT-E images using the described methodology. Finite element models were generated from both *µ*CT and *µ*CT-E set of images keeping all other parameters constant. The stiffness results were compared between *µ*CT and *µ*CT-E models.

### Results

The grayscale values varied almost perfectly linearly with the known density values of the phantom in both imaging facilities (Fig. 4a). There was a perfect linear correlation between grayscale values of both scanning machines (Fig. 4b).

The computational stiffness from the models obtained using *µ*CT-E were over-estimated by about 5% when compared to model generated from *µ*CT images (Table 3).

**Table 3:** Calculated error between FE models based on images from both scanners.

SP #	*µ*CT 100 Stiffness (N/mm)	*µ*CT-E Stiffness (N/mm)	Error (%)
T3CC1	4851	5178	6.74
T2CC1	3800	3896	2.54
T6CC1	4506	4718	4.71

### Discussion

For the equipment used in this study, it was found that a linear relationship could be used to create *µ*CT equivalent images from the HR-pQCT modality provided it was calibrated using the same phantoms.

The error in stiffness values calculated from computer model using *µ*CT-E images were acceptable and the methodology can thus be used to convert *µ*CT equivalent images and FE models based on these images.
